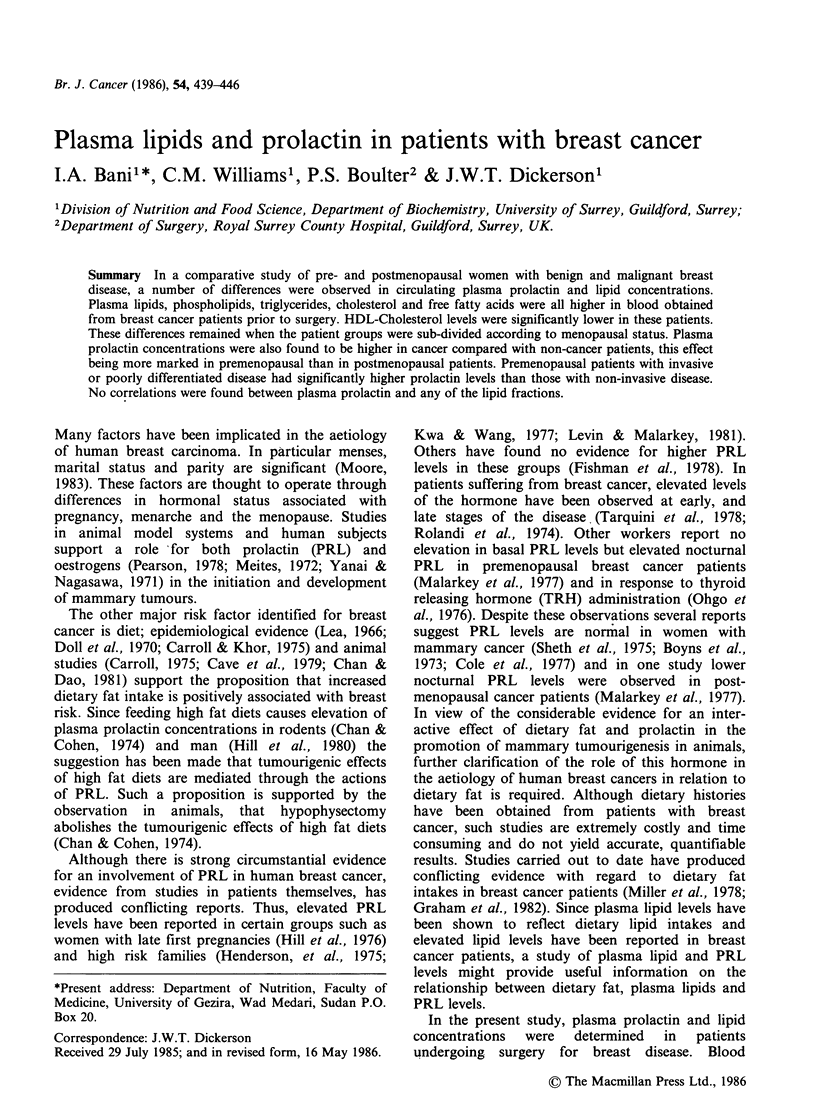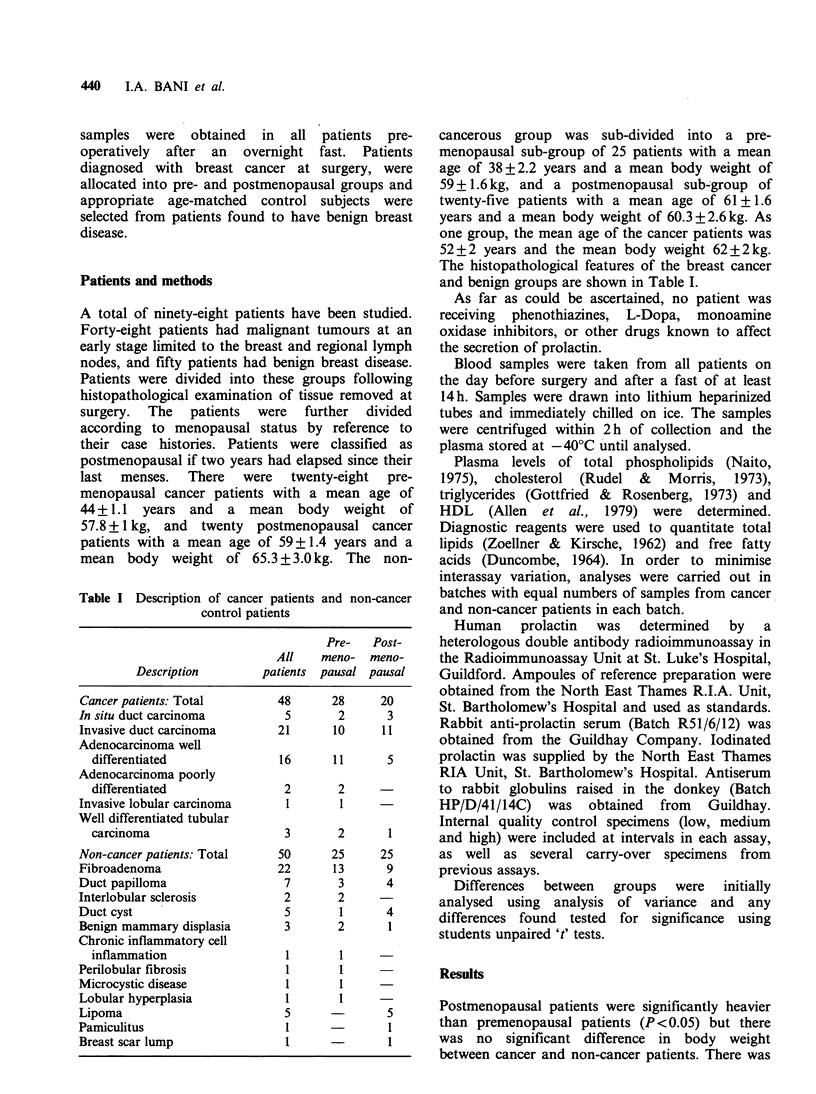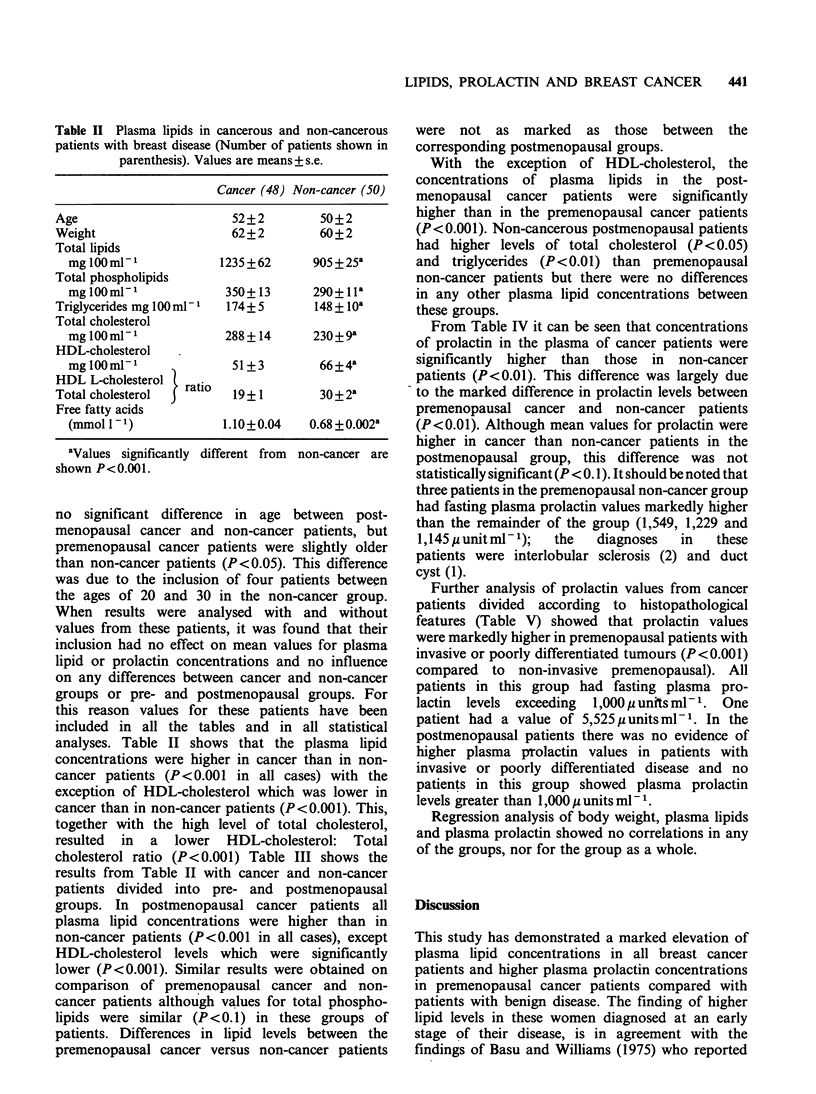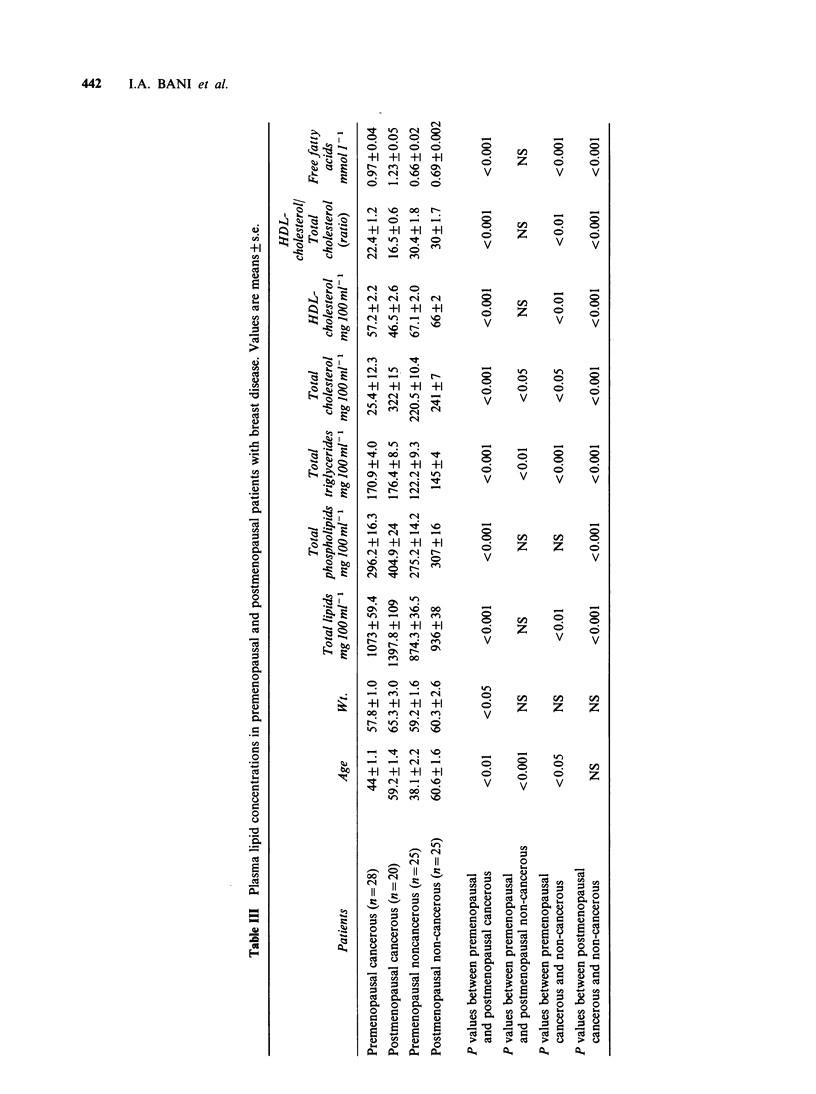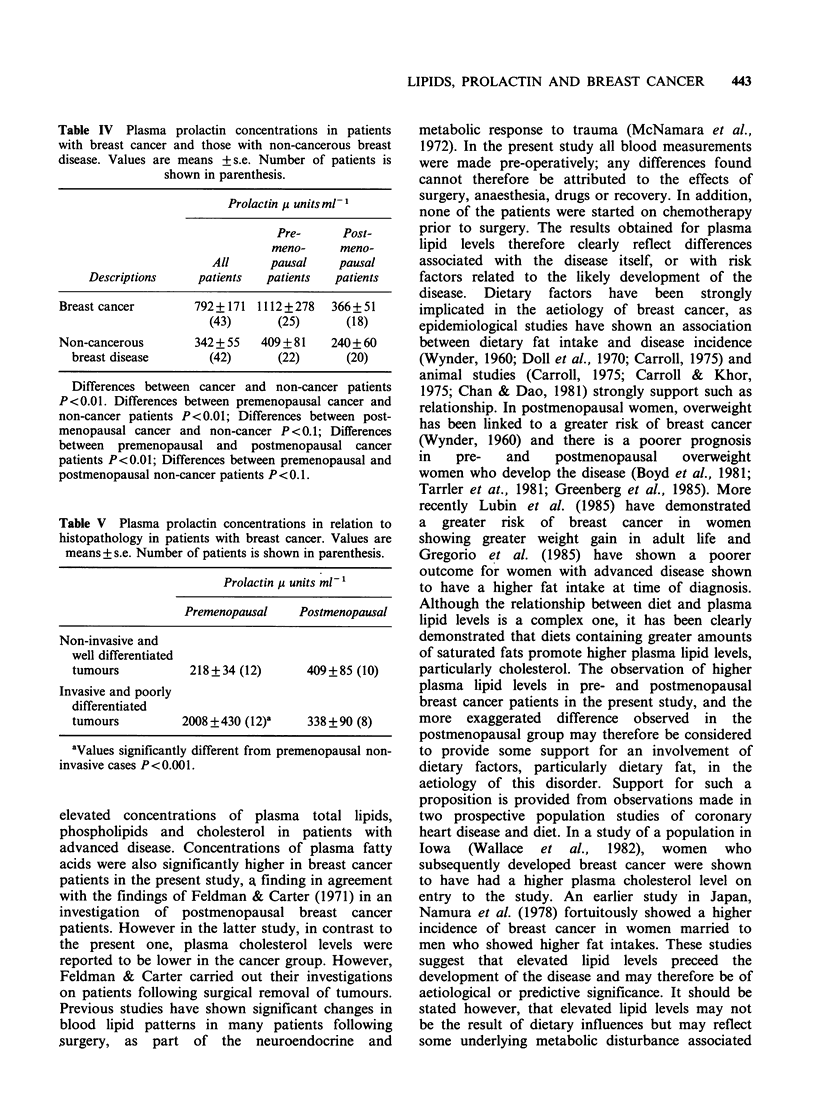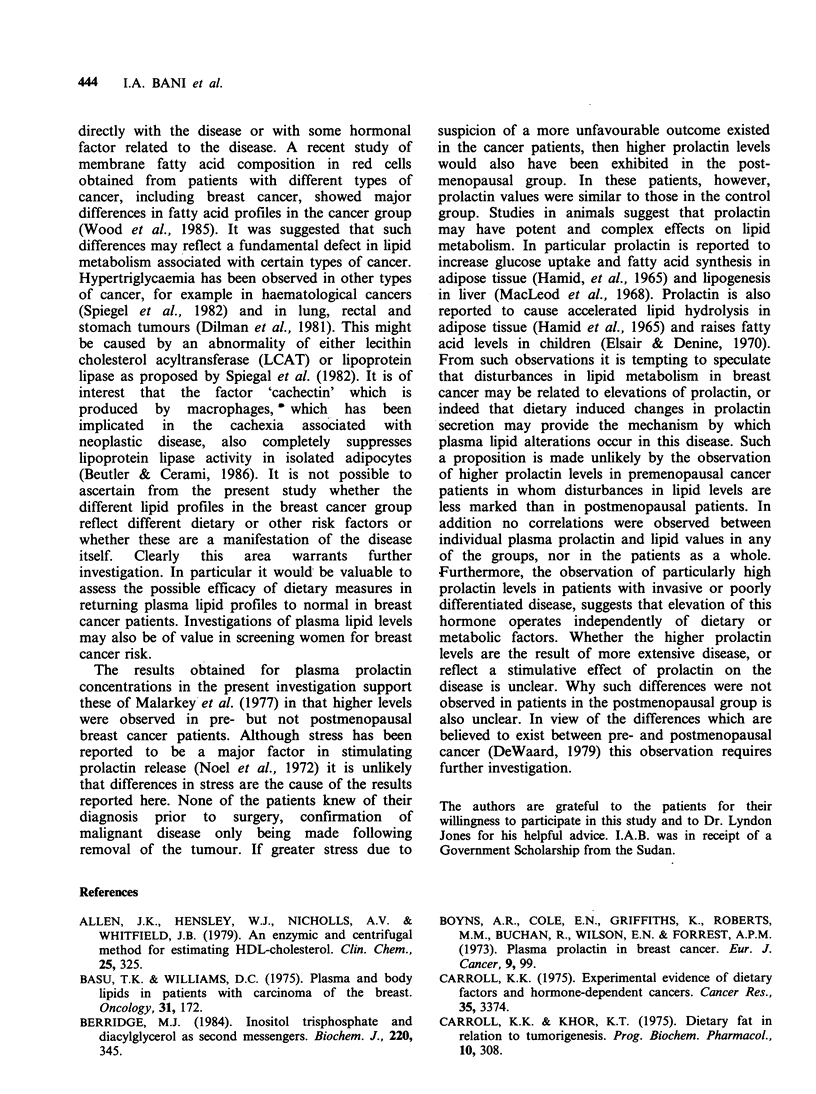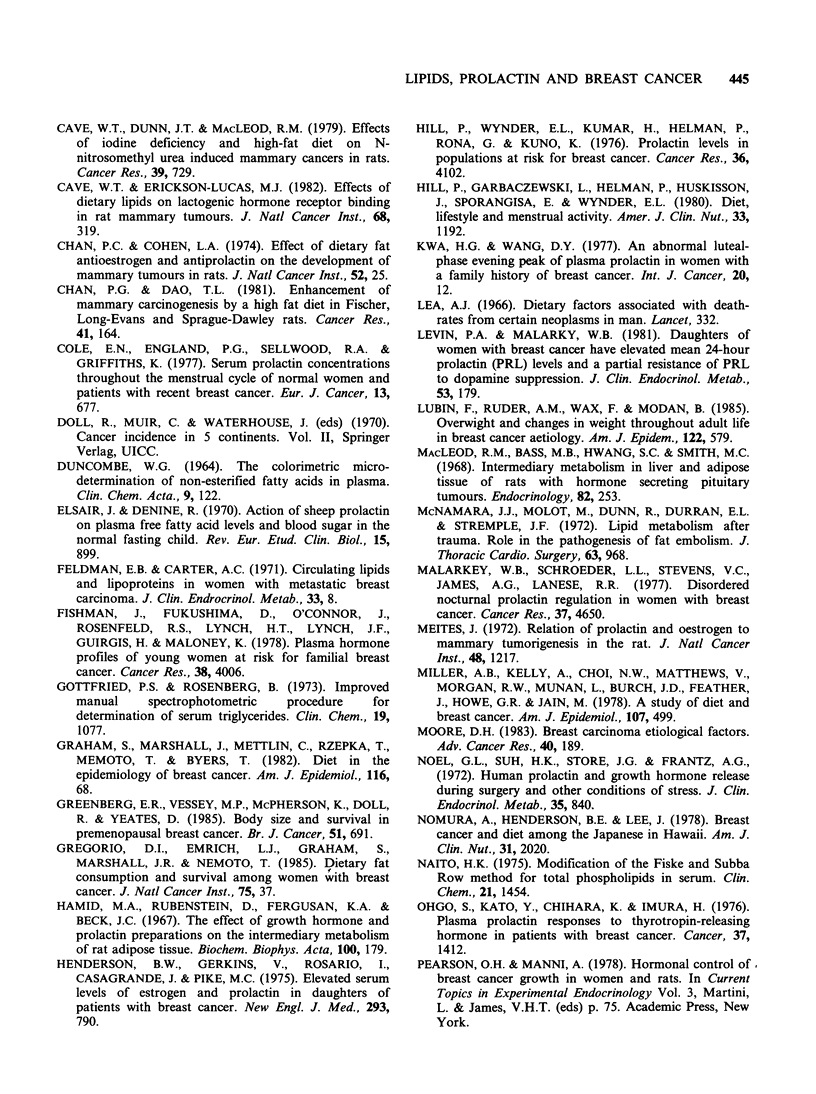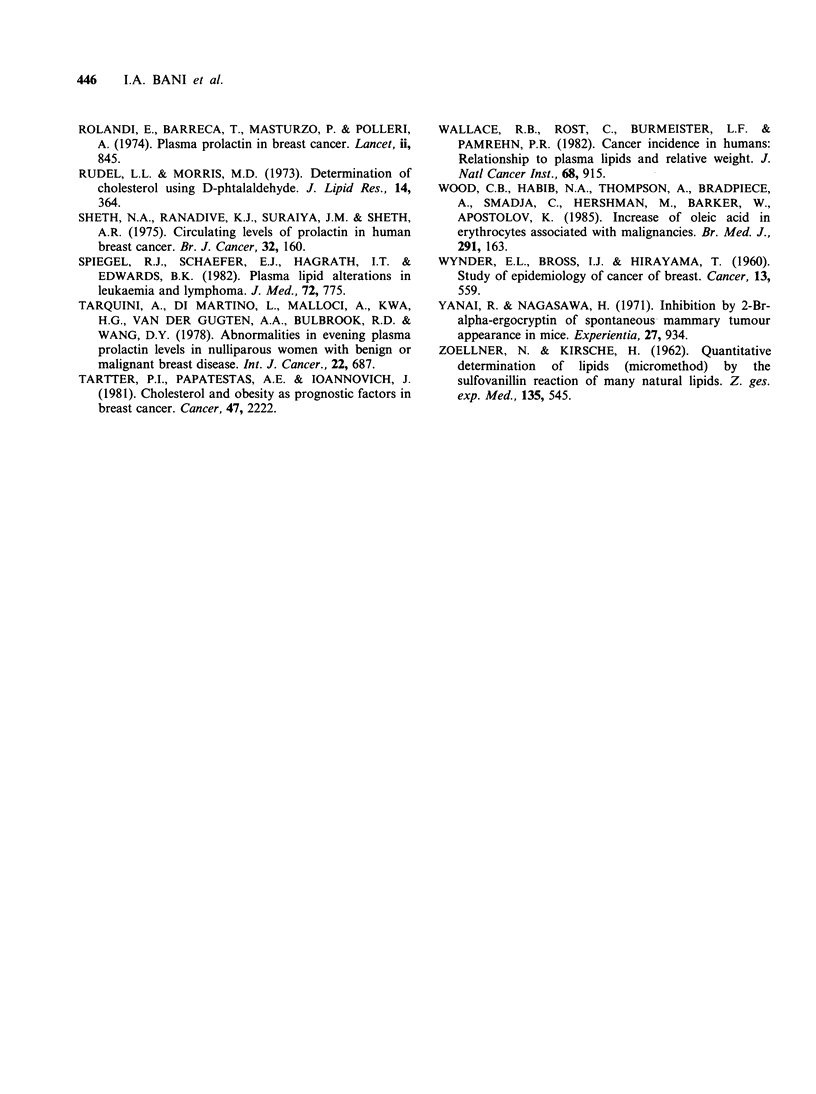# Plasma lipids and prolactin in patients with breast cancer.

**DOI:** 10.1038/bjc.1986.195

**Published:** 1986-09

**Authors:** I. A. Bani, C. M. Williams, P. S. Boulter, J. W. Dickerson

## Abstract

In a comparative study of pre- and postmenopausal women with benign and malignant breast disease, a number of differences were observed in circulating plasma prolactin and lipid concentrations. Plasma lipids, phospholipids, triglycerides, cholesterol and free fatty acids were all higher in blood obtained from breast cancer patients prior to surgery. HDL-Cholesterol levels were significantly lower in these patients. These differences remained when the patient groups were sub-divided according to menopausal status. Plasma prolactin concentrations were also found to be higher in cancer compared with non-cancer patients, this effect being more marked in premenopausal than in postmenopausal patients. Premenopausal patients with invasive or poorly differentiated disease had significantly higher prolactin levels than those with non-invasive disease. No correlations were found between plasma prolactin and any of the lipid fractions.


*Br. J. Cancer* (1986), **54**, 439–446

### Plasma lipids and prolactin in patients with breast cancer

I.A. Bani[1]*, C.M. Williams[1], P.S. Boulter[2] & J.W.T. Dickerson[1]

[1]*Division of Nutrition and Food Science, Department of Biochemistry, University of Surrey, Guildford, Surrey;* [2]*Department of Surgery, Royal Surrey County Hospital, Guildford, Surrey, UK.*

**Summary** In a comparative study of pre- and postmenopausal women with benign and malignant breast disease, a number of differences were observed in circulating plasma prolactin and lipid concentrations. Plasma lipids, phospholipids, triglycerides, cholesterol and free fatty acids were all higher in blood obtained from breast cancer patients prior to surgery. HDL-Cholesterol levels were significantly lower in these patients. These differences remained when the patient groups were sub-divided according to menopausal status. Plasma prolactin concentrations were also found to be higher in cancer compared with non-cancer patients, this effect being more marked in premenopausal than in postmenopausal patients. Premenopausal patients with invasive or poorly differentiated disease had significantly higher prolactin levels than those with non-invasive disease. No correlations were found between plasma prolactin and any of the lipid fractions.

Many factors have been implicated in the aetiology of human breast carcinoma. In particular menses, marital status and parity are significant (Moore, 1983). These factors are thought to operate through differences in hormonal status associated with pregnancy, menarche and the menopause. Studies in animal model systems and human subjects support a role for both prolactin (PRL) and oestrogens (Pearson, 1978; Meites, 1972; Yanai & Nagasawa, 1971) in the initiation and development of mammary tumours.

The other major risk factor identified for breast cancer is diet; epidemiological evidence (Lea, 1966; Doll *et al.*, 1970; Carroll & Khor, 1975) and animal studies (Carroll, 1975; Cave *et al.*, 1979; Chan & Dao, 1981) support the proposition that increased dietary fat intake is positively associated with breast risk. Since feeding high fat diets causes elevation of plasma prolactin concentrations in rodents (Chan & Cohen, 1974) and man (Hill *et al.*, 1980) the suggestion has been made that tumourigenic effects of high fat diets are mediated through the actions of PRL. Such a proposition is supported by the observation in animals, that hypophysectomy abolishes the tumourigenic effects of high fat diets (Chan & Cohen, 1974).

Although there is strong circumstantial evidence for an involvement of PRL in human breast cancer, evidence from studies in patients themselves, has produced conflicting reports. Thus, elevated PRL levels have been reported in certain groups such as women with late first pregnancies (Hill *et al.*, 1976) and high risk families (Henderson, *et al.*, 1975; Kwa & Wang, 1977; Levin & Malarkey, 1981). Others have found no evidence for higher PRL levels in these groups (Fishman *et al.*, 1978). In patients suffering from breast cancer, elevated levels of the hormone have been observed at early, and late stages of the disease (Tarquini *et al.*, 1978; Rolandi *et al.*, 1974). Other workers report no elevation in basal PRL levels but elevated nocturnal PRL in premenopausal breast cancer patients (Malarkey *et al.*, 1977) and in response to thyroid releasing hormone (TRH) administration (Ohgo *et al.*, 1976). Despite these observations several reports suggest PRL levels are normal in women with mammary cancer (Sheth *et al.*, 1975; Boyns *et al.*, 1973; Cole *et al.*, 1977) and in one study lower nocturnal PRL levels were observed in postmenopausal cancer patients (Malarkey *et al.*, 1977). In view of the considerable evidence for an interactive effect of dietary fat and prolactin in the promotion of mammary tumourigenesis in animals, further clarification of the role of this hormone in the aetiology of human breast cancers in relation to dietary fat is required. Although dietary histories have been obtained from patients with breast cancer, such studies are extremely costly and time consuming and do not yield accurate, quantifiable results. Studies carried out to date have produced conflicting evidence with regard to dietary fat intakes in breast cancer patients (Miller *et al.*, 1978; Graham *et al.*, 1982). Since plasma lipid levels have been shown to reflect dietary lipid intakes and elevated lipid levels have been reported in breast cancer patients, a study of plasma lipid and PRL levels might provide useful information on the relationship between dietary fat, plasma lipids and PRL levels.

In the present study, plasma prolactin and lipid concentrations were determined in patients undergoing surgery for breast disease. Blood

*Present address: Department of Nutrition, Faculty of Medicine, University of Gezira, Wad Medari, Sudan P.O. Box 20.

Correspondence: J.W.T. Dickerson
Received 29 July 1985; and in revised form, 16 May 1986.

© The Macmillan Press Ltd., 1986

samples were obtained in all patients pre-operatively after an overnight fast. Patients diagnosed with breast cancer at surgery, were allocated into pre- and postmenopausal groups and appropriate age-matched control subjects were selected from patients found to have benign breast disease.

### Patients and methods

A total of ninety-eight patients have been studied. Forty-eight patients had malignant tumours at an early stage limited to the breast and regional lymph nodes, and fifty patients had benign breast disease. Patients were divided into these groups following histopathological examination of tissue removed at surgery. The patients were further divided according to menopausal status by reference to their case histories. Patients were classified as postmenopausal if two years had elapsed since their last menses. There were twenty-eight pre-menopausal cancer patients with a mean age of $44 \pm 1.1$ years and a mean body weight of $57.8 \pm 1$ kg, and twenty postmenopausal cancer patients with a mean age of $59 \pm 1.4$ years and a mean body weight of $65.3 \pm 3.0$ kg. The non-cancerous group was sub-divided into a pre-menopausal sub-group of 25 patients with a mean age of $38 \pm 2.2$ years and a mean body weight of $59 \pm 1.6$ kg, and a postmenopausal sub-group of twenty-five patients with a mean age of $61 \pm 1.6$ years and a mean body weight of $60.3 \pm 2.6$ kg. As one group, the mean age of the cancer patients was $52 \pm 2$ years and the mean body weight $62 \pm 2$ kg. The histopathological features of the breast cancer and benign groups are shown in Table I.

**Table I** Description of cancer patients and non-cancer control patients

| Description | All patients | Pre-menopausal | Post-menopausal |
|---|---|---|---|
| *Cancer patients:* Total | 48 | 28 | 20 |
| *In situ* duct carcinoma | 5 | 2 | 3 |
| Invasive duct carcinoma | 21 | 10 | 11 |
| Adenocarcinoma well differentiated | 16 | 11 | 5 |
| Adenocarcinoma poorly differentiated | 2 | 2 | — |
| Invasive lobular carcinoma | 1 | 1 | — |
| Well differentiated tubular carcinoma | 3 | 2 | 1 |
| *Non-cancer patients:* Total | 50 | 25 | 25 |
| Fibroadenoma | 22 | 13 | 9 |
| Duct papilloma | 7 | 3 | 4 |
| Interlobular sclerosis | 2 | 2 | — |
| Duct cyst | 5 | 1 | 4 |
| Benign mammary displasia | 3 | 2 | 1 |
| Chronic inflammatory cell inflammation | 1 | 1 | — |
| Perilobular fibrosis | 1 | 1 | — |
| Microcystic disease | 1 | 1 | — |
| Lobular hyperplasia | 1 | 1 | — |
| Lipoma | 5 | — | 5 |
| Pamiculitus | 1 | — | 1 |
| Breast scar lump | 1 | — | 1 |

As far as could be ascertained, no patient was receiving phenothiazines, L-Dopa, monoamine oxidase inhibitors, or other drugs known to affect the secretion of prolactin.

Blood samples were taken from all patients on the day before surgery and after a fast of at least 14 h. Samples were drawn into lithium heparinized tubes and immediately chilled on ice. The samples were centrifuged within 2 h of collection and the plasma stored at $-40$°C until analysed.

Plasma levels of total phospholipids (Naito, 1975), cholesterol (Rudel & Morris, 1973), triglycerides (Gottfried & Rosenberg, 1973) and HDL (Allen *et al.*, 1979) were determined. Diagnostic reagents were used to quantitate total lipids (Zoellner & Kirsche, 1962) and free fatty acids (Duncombe, 1964). In order to minimise interassay variation, analyses were carried out in batches with equal numbers of samples from cancer and non-cancer patients in each batch.

Human prolactin was determined by a heterologous double antibody radioimmunoassay in the Radioimmunoassay Unit at St. Luke's Hospital, Guildford. Ampoules of reference preparation were obtained from the North East Thames R.I.A. Unit, St. Bartholomew's Hospital and used as standards. Rabbit anti-prolactin serum (Batch R51/6/12) was obtained from the Guildhay Company. Iodinated prolactin was supplied by the North East Thames RIA Unit, St. Bartholomew's Hospital. Antiserum to rabbit globulins raised in the donkey (Batch HP/D/41/14C) was obtained from Guildhay. Internal quality control specimens (low, medium and high) were included at intervals in each assay, as well as several carry-over specimens from previous assays.

Differences between groups were initially analysed using analysis of variance and any differences found tested for significance using students unpaired '*t*' tests.

### Results

Postmenopausal patients were significantly heavier than premenopausal patients ($P<0.05$) but there was no significant difference in body weight between cancer and non-cancer patients. There was

**Table II** Plasma lipids in cancerous and non-cancerous patients with breast disease (Number of patients shown in parenthesis). Values are means ± s.e.

| | *Cancer (48)* | *Non-cancer (50)* |
|---|---|---|
| Age | 52±2 | 50±2 |
| Weight | 62±2 | 60±2 |
| Total lipids mg 100 ml$^{-1}$ | 1235±62 | 905±25[a] |
| Total phospholipids mg 100 ml$^{-1}$ | 350±13 | 290±11[a] |
| Triglycerides mg 100 ml$^{-1}$ | 174±5 | 148±10[a] |
| Total cholesterol mg 100 ml$^{-1}$ | 288±14 | 230±9[a] |
| HDL-cholesterol mg 100 ml$^{-1}$ | 51±3 | 66±4[a] |
| HDL L-cholesterol / Total cholesterol ratio | 19±1 | 30±2[a] |
| Free fatty acids (mmol l$^{-1}$) | 1.10±0.04 | 0.68±0.002[a] |

[a]Values significantly different from non-cancer are shown $P<0.001$.

no significant difference in age between postmenopausal cancer and non-cancer patients, but premenopausal cancer patients were slightly older than non-cancer patients ($P<0.05$). This difference was due to the inclusion of four patients between the ages of 20 and 30 in the non-cancer group. When results were analysed with and without values from these patients, it was found that their inclusion had no effect on mean values for plasma lipid or prolactin concentrations and no influence on any differences between cancer and non-cancer groups or pre- and postmenopausal groups. For this reason values for these patients have been included in all the tables and in all statistical analyses. Table II shows that the plasma lipid concentrations were higher in cancer than in non-cancer patients ($P<0.001$ in all cases) with the exception of HDL-cholesterol which was lower in cancer than in non-cancer patients ($P<0.001$). This, together with the high level of total cholesterol, resulted in a lower HDL-cholesterol: Total cholesterol ratio ($P<0.001$) Table III shows the results from Table II with cancer and non-cancer patients divided into pre- and postmenopausal groups. In postmenopausal cancer patients all plasma lipid concentrations were higher than in non-cancer patients ($P<0.001$ in all cases), except HDL-cholesterol levels which were significantly lower ($P<0.001$). Similar results were obtained on comparison of premenopausal cancer and non-cancer patients although values for total phospholipids were similar ($P<0.1$) in these groups of patients. Differences in lipid levels between the premenopausal cancer versus non-cancer patients were not as marked as those between the corresponding postmenopausal groups.

With the exception of HDL-cholesterol, the concentrations of plasma lipids in the postmenopausal cancer patients were significantly higher than in the premenopausal cancer patients ($P<0.001$). Non-cancerous postmenopausal patients had higher levels of total cholesterol ($P<0.05$) and triglycerides ($P<0.01$) than premenopausal non-cancer patients but there were no differences in any other plasma lipid concentrations between these groups.

From Table IV it can be seen that concentrations of prolactin in the plasma of cancer patients were significantly higher than those in non-cancer patients ($P<0.01$). This difference was largely due to the marked difference in prolactin levels between premenopausal cancer and non-cancer patients ($P<0.01$). Although mean values for prolactin were higher in cancer than non-cancer patients in the postmenopausal group, this difference was not statistically significant ($P<0.1$). It should be noted that three patients in the premenopausal non-cancer group had fasting plasma prolactin values markedly higher than the remainder of the group (1,549, 1,229 and 1,145 $\mu$unit ml$^{-1}$); the diagnoses in these patients were interlobular sclerosis (2) and duct cyst (1).

Further analysis of prolactin values from cancer patients divided according to histopathological features (Table V) showed that prolactin values were markedly higher in premenopausal patients with invasive or poorly differentiated tumours ($P<0.001$) compared to non-invasive premenopausal). All patients in this group had fasting plasma prolactin levels exceeding 1,000 $\mu$units ml$^{-1}$. One patient had a value of 5,525 $\mu$units ml$^{-1}$. In the postmenopausal patients there was no evidence of higher plasma prolactin values in patients with invasive or poorly differentiated disease and no patients in this group showed plasma prolactin levels greater than 1,000 $\mu$units ml$^{-1}$.

Regression analysis of body weight, plasma lipids and plasma prolactin showed no correlations in any of the groups, nor for the group as a whole.

### Discussion

This study has demonstrated a marked elevation of plasma lipid concentrations in all breast cancer patients and higher plasma prolactin concentrations in premenopausal cancer patients compared with patients with benign disease. The finding of higher lipid levels in these women diagnosed at an early stage of their disease, is in agreement with the findings of Basu and Williams (1975) who reported

**Table III** Plasma lipid concentrations in premenopausal and postmenopausal patients with breast disease. Values are means ± s.e.

| *Patients* | *Age* | *Wt.* | *Total lipids mg 100 ml$^{-1}$* | *Total phospholipids mg 100 ml$^{-1}$* | *Total triglycerides mg 100 ml$^{-1}$* | *Total cholesterol mg 100 ml$^{-1}$* | *HDL-cholesterol mg 100 ml$^{-1}$* | *HDL-cholesterol/Total cholesterol (ratio)* | *Free fatty acids mmol l$^{-1}$* |
|---|---|---|---|---|---|---|---|---|---|
| Premenopausal cancerous (*n* = 28) | 44 ± 1.1 | 57.8 ± 1.0 | 1073 ± 59.4 | 296.2 ± 16.3 | 170.9 ± 4.0 | 25.4 ± 12.3 | 57.2 ± 2.2 | 22.4 ± 1.2 | 0.97 ± 0.04 |
| Postmenopausal cancerous (*n* = 20) | 59.2 ± 1.4 | 65.3 ± 3.0 | 1397.8 ± 109 | 404.9 ± 24 | 176.4 ± 8.5 | 322 ± 15 | 46.5 ± 2.6 | 16.5 ± 0.6 | 1.23 ± 0.05 |
| Premenopausal noncancerous (*n* = 25) | 38.1 ± 2.2 | 59.2 ± 1.6 | 874.3 ± 36.5 | 275.2 ± 14.2 | 122.2 ± 9.3 | 220.5 ± 10.4 | 67.1 ± 2.0 | 30.4 ± 1.8 | 0.66 ± 0.02 |
| Postmenopausal non-cancerous (*n* = 25) | 60.6 ± 1.6 | 60.3 ± 2.6 | 936 ± 38 | 307 ± 16 | 145 ± 4 | 241 ± 7 | 66 ± 2 | 30 ± 1.7 | 0.69 ± 0.002 |
| *P* values between premenopausal and postmenopausal cancerous | < 0.01 | < 0.05 | < 0.001 | < 0.001 | < 0.001 | < 0.001 | < 0.001 | < 0.001 | < 0.001 |
| *P* values between premenopausal and postmenopausal non-cancerous | < 0.001 | NS | NS | NS | < 0.01 | < 0.05 | NS | NS | NS |
| *P* values between premenopausal cancerous and non-cancerous | < 0.05 | NS | < 0.01 | NS | < 0.001 | < 0.05 | < 0.01 | < 0.01 | < 0.001 |
| *P* values between postmenopausal cancerous and non-cancerous | NS | NS | < 0.001 | < 0.001 | < 0.001 | < 0.001 | < 0.001 | < 0.001 | < 0.001 |

**Table IV** Plasma prolactin concentrations in patients with breast cancer and those with non-cancerous breast disease. Values are means ±s.e. Number of patients is shown in parenthesis.

| | *Prolactin μ units ml⁻¹* | | |
|---|---|---|---|
| *Descriptions* | *All patients* | *Pre-meno-pausal patients* | *Post-meno-pausal patients* |
| Breast cancer | 792±171 (43) | 1112±278 (25) | 366±51 (18) |
| Non-cancerous breast disease | 342±55 (42) | 409±81 (22) | 240±60 (20) |

Differences between cancer and non-cancer patients $P<0.01$. Differences between premenopausal cancer and non-cancer patients $P<0.01$; Differences between postmenopausal cancer and non-cancer $P<0.1$; Differences between premenopausal and postmenopausal cancer patients $P<0.01$; Differences between premenopausal and postmenopausal non-cancer patients $P<0.1$.

**Table V** Plasma prolactin concentrations in relation to histopathology in patients with breast cancer. Values are means±s.e. Number of patients is shown in parenthesis.

| | *Prolactin μ units ml⁻¹* | |
|---|---|---|
| | *Premenopausal* | *Postmenopausal* |
| Non-invasive and well differentiated tumours | 218±34 (12) | 409±85 (10) |
| Invasive and poorly differentiated tumours | 2008±430 (12)[a] | 338±90 (8) |

[a]Values significantly different from premenopausal non-invasive cases $P<0.001$.

elevated concentrations of plasma total lipids, phospholipids and cholesterol in patients with advanced disease. Concentrations of plasma fatty acids were also significantly higher in breast cancer patients in the present study, a finding in agreement with the findings of Feldman & Carter (1971) in an investigation of postmenopausal breast cancer patients. However in the latter study, in contrast to the present one, plasma cholesterol levels were reported to be lower in the cancer group. However, Feldman & Carter carried out their investigations on patients following surgical removal of tumours. Previous studies have shown significant changes in blood lipid patterns in many patients following surgery, as part of the neuroendocrine and metabolic response to trauma (McNamara *et al.*, 1972). In the present study all blood measurements were made pre-operatively; any differences found cannot therefore be attributed to the effects of surgery, anaesthesia, drugs or recovery. In addition, none of the patients were started on chemotherapy prior to surgery. The results obtained for plasma lipid levels therefore clearly reflect differences associated with the disease itself, or with risk factors related to the likely development of the disease. Dietary factors have been strongly implicated in the aetiology of breast cancer, as epidemiological studies have shown an association between dietary fat intake and disease incidence (Wynder, 1960; Doll *et al.*, 1970; Carroll, 1975) and animal studies (Carroll, 1975; Carroll & Khor, 1975; Chan & Dao, 1981) strongly support such as relationship. In postmenopausal women, overweight has been linked to a greater risk of breast cancer (Wynder, 1960) and there is a poorer prognosis in pre- and postmenopausal overweight women who develop the disease (Boyd *et al.*, 1981; Tarrler *et at.*, 1981; Greenberg *et al.*, 1985). More recently Lubin *et al.* (1985) have demonstrated a greater risk of breast cancer in women showing greater weight gain in adult life and Gregorio *et al.* (1985) have shown a poorer outcome for women with advanced disease shown to have a higher fat intake at time of diagnosis. Although the relationship between diet and plasma lipid levels is a complex one, it has been clearly demonstrated that diets containing greater amounts of saturated fats promote higher plasma lipid levels, particularly cholesterol. The observation of higher plasma lipid levels in pre- and postmenopausal breast cancer patients in the present study, and the more exaggerated difference observed in the postmenopausal group may therefore be considered to provide some support for an involvement of dietary factors, particularly dietary fat, in the aetiology of this disorder. Support for such a proposition is provided from observations made in two prospective population studies of coronary heart disease and diet. In a study of a population in Iowa (Wallace *et al.*, 1982), women who subsequently developed breast cancer were shown to have had a higher plasma cholesterol level on entry to the study. An earlier study in Japan, Namura *et al.* (1978) fortuitously showed a higher incidence of breast cancer in women married to men who showed higher fat intakes. These studies suggest that elevated lipid levels preceed the development of the disease and may therefore be of aetiological or predictive significance. It should be stated however, that elevated lipid levels may not be the result of dietary influences but may reflect some underlying metabolic disturbance associated directly with the disease or with some hormonal factor related to the disease. A recent study of membrane fatty acid composition in red cells obtained from patients with different types of cancer, including breast cancer, showed major differences in fatty acid profiles in the cancer group (Wood *et al.*, 1985). It was suggested that such differences may reflect a fundamental defect in lipid metabolism associated with certain types of cancer. Hypertriglycaemia has been observed in other types of cancer, for example in haematological cancers (Spiegel *et al.*, 1982) and in lung, rectal and stomach tumours (Dilman *et al.*, 1981). This might be caused by an abnormality of either lecithin cholesterol acyltransferase (LCAT) or lipoprotein lipase as proposed by Spiegal *et al.* (1982). It is of interest that the factor 'cachectin' which is produced by macrophages, which has been implicated in the cachexia associated with neoplastic disease, also completely suppresses lipoprotein lipase activity in isolated adipocytes (Beutler & Cerami, 1986). It is not possible to ascertain from the present study whether the different lipid profiles in the breast cancer group reflect different dietary or other risk factors or whether these are a manifestation of the disease itself. Clearly this area warrants further investigation. In particular it would be valuable to assess the possible efficacy of dietary measures in returning plasma lipid profiles to normal in breast cancer patients. Investigations of plasma lipid levels may also be of value in screening women for breast cancer risk.

The results obtained for plasma prolactin concentrations in the present investigation support these of Malarkey *et al.* (1977) in that higher levels were observed in pre- but not postmenopausal breast cancer patients. Although stress has been reported to be a major factor in stimulating prolactin release (Noel *et al.*, 1972) it is unlikely that differences in stress are the cause of the results reported here. None of the patients knew of their diagnosis prior to surgery, confirmation of malignant disease only being made following removal of the tumour. If greater stress due to suspicion of a more unfavourable outcome existed in the cancer patients, then higher prolactin levels would also have been exhibited in the postmenopausal group. In these patients, however, prolactin values were similar to those in the control group. Studies in animals suggest that prolactin may have potent and complex effects on lipid metabolism. In particular prolactin is reported to increase glucose uptake and fatty acid synthesis in adipose tissue (Hamid, *et al.*, 1965) and lipogenesis in liver (MacLeod *et al.*, 1968). Prolactin is also reported to cause accelerated lipid hydrolysis in adipose tissue (Hamid *et al.*, 1965) and raises fatty acid levels in children (Elsair & Denine, 1970). From such observations it is tempting to speculate that disturbances in lipid metabolism in breast cancer may be related to elevations of prolactin, or indeed that dietary induced changes in prolactin secretion may provide the mechanism by which plasma lipid alterations occur in this disease. Such a proposition is made unlikely by the observation of higher prolactin levels in premenopausal cancer patients in whom disturbances in lipid levels are less marked than in postmenopausal patients. In addition no correlations were observed between individual plasma prolactin and lipid values in any of the groups, nor in the patients as a whole. Furthermore, the observation of particularly high prolactin levels in patients with invasive or poorly differentiated disease, suggests that elevation of this hormone operates independently of dietary or metabolic factors. Whether the higher prolactin levels are the result of more extensive disease, or reflect a stimulative effect of prolactin on the disease is unclear. Why such differences were not observed in patients in the postmenopausal group is also unclear. In view of the differences which are believed to exist between pre- and postmenopausal cancer (DeWaard, 1979) this observation requires further investigation.

The authors are grateful to the patients for their willingness to participate in this study and to Dr. Lyndon Jones for his helpful advice. I.A.B. was in receipt of a Government Scholarship from the Sudan.

### References

ALLEN, J.K., HENSLEY, W.J., NICHOLLS, A.V. & WHITFIELD, J.B. (1979). An enzymic and centrifugal method for estimating HDL-cholesterol. *Clin. Chem.*, **25**, 325.

BASU, T.K. & WILLIAMS, D.C. (1975). Plasma and body lipids in patients with carcinoma of the breast. *Oncology*, **31**, 172.

BERRIDGE, M.J. (1984). Inositol trisphosphate and diacylglycerol as second messengers. *Biochem. J.*, **220**, 345.

BOYNS, A.R., COLE, E.N., GRIFFITHS, K., ROBERTS, M.M., BUCHAN, R., WILSON, E.N. & FORREST, A.P.M. (1973). Plasma prolactin in breast cancer. *Eur. J. Cancer*, **9**, 99.

CARROLL, K.K. (1975). Experimental evidence of dietary factors and hormone-dependent cancers. *Cancer Res.*, **35**, 3374.

CARROLL, K.K. & KHOR, K.T. (1975). Dietary fat in relation to tumorigenesis. *Prog. Biochem. Pharmacol.*, **10**, 308.

CAVE, W.T., DUNN, J.T. & MACLEOD, R.M. (1979). Effects of iodine deficiency and high-fat diet on N-nitrosomethyl urea induced mammary cancers in rats. *Cancer Res.*, **39**, 729.

CAVE, W.T. & ERICKSON-LUCAS, M.J. (1982). Effects of dietary lipids on lactogenic hormone receptor binding in rat mammary tumours. *J. Natl Cancer Inst.*, **68**, 319.

CHAN, P.C. & COHEN, L.A. (1974). Effect of dietary fat antioestrogen and antiprolactin on the development of mammary tumours in rats. *J. Natl Cancer Inst.*, **52**, 25.

CHAN, P.G. & DAO, T.L. (1981). Enhancement of mammary carcinogenesis by a high fat diet in Fischer, Long-Evans and Sprague-Dawley rats. *Cancer Res.*, **41**, 164.

COLE, E.N., ENGLAND, P.G., SELLWOOD, R.A. & GRIFFITHS, K. (1977). Serum prolactin concentrations throughout the menstrual cycle of normal women and patients with recent breast cancer. *Eur. J. Cancer*, **13**, 677.

DOLL, R., MUIR, C. & WATERHOUSE, J. (eds) (1970). Cancer incidence in 5 continents. Vol. II, Springer Verlag, UICC.

DUNCOMBE, W.G. (1964). The colorimetric micro-determination of non-esterified fatty acids in plasma. *Clin. Chem. Acta.*, **9**, 122.

ELSAIR, J. & DENINE, R. (1970). Action of sheep prolactin on plasma free fatty acid levels and blood sugar in the normal fasting child. *Rev. Eur. Etud. Clin. Biol.*, **15**, 899.

FELDMAN, E.B. & CARTER, A.C. (1971). Circulating lipids and lipoproteins in women with metastatic breast carcinoma. *J. Clin. Endrocrinol. Metab.*, **33**, 8.

FISHMAN, J., FUKUSHIMA, D., O'CONNOR, J., ROSENFELD, R.S., LYNCH, H.T., LYNCH, J.F., GUIRGIS, H. & MALONEY, K. (1978). Plasma hormone profiles of young women at risk for familial breast cancer. *Cancer Res.*, **38**, 4006.

GOTTFRIED, P.S. & ROSENBERG, B. (1973). Improved manual spectrophotometric procedure for determination of serum triglycerides. *Clin. Chem.*, **19**, 1077.

GRAHAM, S., MARSHALL, J., METTLIN, C., RZEPKA, T., MEMOTO, T. & BYERS, T. (1982). Diet in the epidemiology of breast cancer. *Am. J. Epidemiol.*, **116**, 68.

GREENBERG, E.R., VESSEY, M.P., MCPHERSON, K., DOLL, R. & YEATES, D. (1985). Body size and survival in premenopausal breast cancer. *Br. J. Cancer*, **51**, 691.

GREGORIO, D.I., EMRICH, L.J., GRAHAM, S., MARSHALL, J.R. & NEMOTO, T. (1985). Dietary fat consumption and survival among women with breast cancer. *J. Natl Cancer Inst.*, **75**, 37.

HAMID, M.A., RUBENSTEIN, D., FERGUSAN, K.A. & BECK, J.C. (1967). The effect of growth hormone and prolactin preparations on the intermediary metabolism of rat adipose tissue. *Biochem. Biophys. Acta*, **100**, 179.

HENDERSON, B.W., GERKINS, V., ROSARIO, I., CASAGRANDE, J. & PIKE, M.C. (1975). Elevated serum levels of estrogen and prolactin in daughters of patients with breast cancer. *New Engl. J. Med.*, **293**, 790.

HILL, P., WYNDER, E.L., KUMAR, H., HELMAN, P., RONA, G. & KUNO, K. (1976). Prolactin levels in populations at risk for breast cancer. *Cancer Res.*, **36**, 4102.

HILL, P., GARBACZEWSKI, L., HELMAN, P., HUSKISSON, J., SPORANGISA, E. & WYNDER, E.L. (1980). Diet, lifestyle and menstrual activity. *Amer. J. Clin. Nut.*, **33**, 1192.

KWA, H.G. & WANG, D.Y. (1977). An abnormal luteal-phase evening peak of plasma prolactin in women with a family history of breast cancer. *Int. J. Cancer*, **20**, 12.

LEA, A.J. (1966). Dietary factors associated with death-rates from certain neoplasms in man. *Lancet*, 332.

LEVIN, P.A. & MALARKY, W.B. (1981). Daughters of women with breast cancer have elevated mean 24-hour prolactin (PRL) levels and a partial resistance of PRL to dopamine suppression. *J. Clin. Endocrinol. Metab.*, **53**, 179.

LUBIN, F., RUDER, A.M., WAX, F. & MODAN, B. (1985). Overwight and changes in weight throughout adult life in breast cancer aetiology. *Am. J. Epidem.*, **122**, 579.

MACLEOD, R.M., BASS, M.B., HWANG, S.C. & SMITH, M.C. (1968). Intermediary metabolism in liver and adipose tissue of rats with hormone secreting pituitary tumours. *Endocrinology*, **82**, 253.

MCNAMARA, J.J., MOLOT, M., DUNN, R., DURRAN, E.L. & STREMPLE, J.F. (1972). Lipid metabolism after trauma. Role in the pathogenesis of fat embolism. *J. Thoracic Cardio. Surgery*, **63**, 968.

MALARKEY, W.B., SCHROEDER, L.L., STEVENS, V.C., JAMES, A.G., LANESE, R.R. (1977). Disordered nocturnal prolactin regulation in women with breast cancer. *Cancer Res.*, **37**, 4650.

MEITES, J. (1972). Relation of prolactin and oestrogen to mammary tumorigenesis in the rat. *J. Natl Cancer Inst.*, **48**, 1217.

MILLER, A.B., KELLY, A., CHOI, N.W., MATTHEWS, V., MORGAN, R.W., MUNAN, L., BURCH, J.D., FEATHER, J., HOWE, G.R. & JAIN, M. (1978). A study of diet and breast cancer. *Am. J. Epidemiol.*, **107**, 499.

MOORE, D.H. (1983). Breast carcinoma etiological factors. *Adv. Cancer Res.*, **40**, 189.

NOEL, G.L., SUH, H.K., STORE, J.G. & FRANTZ, A.G., (1972). Human prolactin and growth hormone release during surgery and other conditions of stress. *J. Clin. Endocrinol. Metab.*, **35**, 840.

NOMURA, A., HENDERSON, B.E. & LEE, J. (1978). Breast cancer and diet among the Japanese in Hawaii. *Am. J. Clin. Nut.*, **31**, 2020.

NAITO, H.K. (1975). Modification of the Fiske and Subba Row method for total phospholipids in serum. *Clin. Chem.*, **21**, 1454.

OHGO, S., KATO, Y., CHIHARA, K. & IMURA, H. (1976). Plasma prolactin responses to thyrotropin-releasing hormone in patients with breast cancer. *Cancer*, **37**, 1412.

PEARSON, O.H. & MANNI, A. (1978). Hormonal control of breast cancer growth in women and rats. In *Current Topics in Experimental Endocrinology* Vol. 3, Martini, L. & James, V.H.T. (eds) p. 75. Academic Press, New York.

ROLANDI, E., BARRECA, T., MASTURZO, P. & POLLERI, A. (1974). Plasma prolactin in breast cancer. *Lancet*, **ii**, 845.

RUDEL, L.L. & MORRIS, M.D. (1973). Determination of cholesterol using D-phtalaldehyde. *J. Lipid Res.*, **14**, 364.

SHETH, N.A., RANADIVE, K.J., SURAIYA, J.M. & SHETH, A.R. (1975). Circulating levels of prolactin in human breast cancer. *Br. J. Cancer*, **32**, 160.

SPIEGEL, R.J., SCHAEFER, E.J., HAGRATH, I.T. & EDWARDS, B.K. (1982). Plasma lipid alterations in leukaemia and lymphoma. *J. Med.*, **72**, 775.

TARQUINI, A., DI MARTINO, L., MALLOCI, A., KWA, H.G., VAN DER GUGTEN, A.A., BULBROOK, R.D. & WANG, D.Y. (1978). Abnormalities in evening plasma prolactin levels in nulliparous women with benign or malignant breast disease. *Int. J. Cancer.*, **22**, 687.

TARTTER, P.I., PAPATESTAS, A.E. & IOANNOVICH, J. (1981). Cholesterol and obesity as prognostic factors in breast cancer. *Cancer*, **47**, 2222.

WALLACE, R.B., ROST, C., BURMEISTER, L.F. & PAMREHN, P.R. (1982). Cancer incidence in humans: Relationship to plasma lipids and relative weight. *J. Natl Cancer Inst.*, **68**, 915.

WOOD, C.B., HABIB, N.A., THOMPSON, A., BRADPIECE, A., SMADJA, C., HERSHMAN, M., BARKER, W., APOSTOLOV, K. (1985). Increase of oleic acid in erythrocytes associated with malignancies. *Br. Med. J.*, **291**, 163.

WYNDER, E.L., BROSS, I.J. & HIRAYAMA, T. (1960). Study of epidemiology of cancer of breast. *Cancer*, **13**, 559.

YANAI, R. & NAGASAWA, H. (1971). Inhibition by 2-Br-alpha-ergocryptin of spontaneous mammary tumour appearance in mice. *Experientia*, **27**, 934.

ZOELLNER, N. & KIRSCHE, H. (1962). Quantitative determination of lipids (micromethod) by the sulfovanillin reaction of many natural lipids. *Z. ges. exp. Med.*, **135**, 545.